# Transcultural adaptation and psychometric evaluation of the Chinese version of attitude and confidence with oral healthcare among nursing students scale: a cross-sectional survey in China

**DOI:** 10.3389/fpubh.2025.1689136

**Published:** 2025-11-25

**Authors:** Yaqin Li, Chengyang Xie, Yan Yue, Weihao Kong, Yuexuan Ma, Kefan Chen, Biyao Liu, Xiaoli Liao, Lingli Li

**Affiliations:** 1 West China Hospital, Sichuan University/West China School of Nursing, Sichuan University, Chengdu, China; 2 School of Nursing, The Hong Kong Polytechnic University, Hong Kong, Hong Kong SAR, China; 3 School of Nursing, The Sun Yat-sen University, Guangzhou, China; 4 Nursing Key Laboratory of Sichuan Province, Chengdu, China

**Keywords:** transcultural adaptation, psychometric evaluation, attitude, confidence, oral healthcare, nursing students

## Abstract

**Objective:**

Positive attitudes and strong confidence toward oral healthcare among nursing students are essential prerequisites for providing quality oral healthcare, which directly impacts the likelihood and effectiveness of their post-training care practices. However, no validated scale is currently available in China to measure nursing students’ attitudes toward oral healthcare and confidence. This study aims to adapt the original English version of the attitude and confidence with oral healthcare among nursing students (ACORN) scale into Chinese culture and assess its psychometric properties.

**Methods:**

This study followed the Brislin Translation Model, including forward translation, back translation, and expert consultation (consultation of the differences between the original English version and the synthesized back-translated version of the ACORN scale with the original author, pilot testing, expert consultation, and bilingual testing). The formal experiment was conducted across eight universities offering bachelor’s nursing programs in China (Chengdu and Shenyang City), involving 536 nursing students who had completed oral healthcare courses. Item analysis, exploratory factor analysis (EFA), confirmatory factor analysis (CFA), and reliability testing (internal consistency and test–retest reliability) were conducted. The data were divided into two equal sets using the random number table function in SPSS, one for EFA and the other for CFA.

**Results:**

The EFA extracted three dimensions: attitudes toward oral care (8 items), confidence in oral care practices (4 items), and confidence in diagnosing oral diseases (10 items), with a cumulative variance contribution rate of 66.33%. The CFA confirmed the three-factor structure with excellent model fit indicators. The overall Cronbach’s alpha for the Chinese version of the ACORN scale was 0.95, with the three dimensions having values of Cronbach’s alphas of 0.92, 0.85, and 0.94, respectively. Twenty nursing students participated in a test–retest survey after a two-week interval, resulting in a test–retest value of 0.76.

**Conclusion:**

The Chinese version of the ACORN scale exhibits strong reliability and validity performances and can be used to assess the oral care-related attitudes and confidence of nursing students in China.

## 1 Introduction

Oral health refers to the integrity and well-being status of the mouth, teeth, and orofacial structures that enable individuals to perform essential physiological functions (e.g., eating, breathing, and speaking) (1), as well as prompting psychosocial functions (e.g., decreasing discomfort/embarrassment in daily life and enhancing confidence in socialization) (1). To be specific, the oral cavity is a prominent facial feature that significantly shapes an individual’s self-image and self-perception through both its appearance and function (2). Oral diseases frequently impair dental aesthetics or cause bad breath (3, 4), which can lead to people’s dissatisfaction with their appearance and, consequently, diminish their self-esteem and social confidence (5–9). Moreover, many oral conditions involve functional limitations and chronic pain, substantially reducing patients’ quality of life by interfering with essential activities such as eating, long-term nutrition, and sleep (10–12). Periodontal disease and dental caries pose the most significant oral disease burden globally (13). Research indicates that periodontal disease affects over half of the world’s population, with severe cases accounting for 10.5 to 12.0% of this group (14). Similarly, dental caries in permanent teeth remains highly prevalent, affecting approximately 33.7 to 37.3% of people globally (15). Research consistently shows that poor oral hygiene is a primary cause of oral diseases (e.g., dental caries and periodontal disease). A systematic review and meta-analysis of 56 studies established that inadequate oral hygiene and plaque accumulation are significant drivers of periodontitis. The analysis found that the risk of developing periodontitis is two to five times higher in individuals with moderate-to-poor oral hygiene than in those with better hygiene (16). A survey of 250 preschool and primary school children linked irregular brushing, the practice of finger brushing, and a lack of parental supervision to a higher incidence of untreated cavities and their subsequent complications (17). The implications of these oral conditions extend beyond the mouth, as they were recognized as risk factors for serious systemic diseases. A joint symposium by the European Federation of Periodontology and the World Organization of Family Doctors (Europe) has established consensus: periodontitis is an independent risk factor for several systemic conditions, including cardiovascular disease, diabetes, chronic obstructive pulmonary disease, obstructive sleep apnea, and COVID-19 complications (18). Furthermore, one systematic review of studies in patients aged 65 and older found a link between periodontal disease and Alzheimer’s disease, suggesting that treating periodontal disease could be a potential strategy for Alzheimer’s prevention (19). Similarly, another systematic review of 50 studies indicated a positive correlation between periodontal disease and an increased risk of several cancers, including oral, lung, and pancreatic cancer (20).

Regular high-quality oral healthcare can not only prevent/decrease the prevalence of oral diseases, relieve patient suffering (21), improve their quality of life (22), and enhance their holistic well-being (23). Nurses/future nurses are the first-line oral health defenders of the whole society, including assessing oral status, providing regular oral health education, and delivering high-quality oral healthcare (24–26), as vital components included in their daily work routines in various clinical settings, including clinical wards (24, 25), communities (26), and long-term facilities (27). According to the Knowledge, Attitude/Belief, Practice (KAP) theory, human behavior change involves three consecutive processes: acquiring knowledge, forming beliefs/attitudes, and shaping behavior (28). Besides obtaining knowledge/skills, according to the Social Cognitive Theory (SCT) (29, 30), the quality of future nursing practice mostly results from the interplay among nursing students’ personal factors, including physiological aspects (e.g., race, gender, body height), psychological attributes (e.g., cognition, attitude, confidence), environmental factors (e.g., social supports/barriers, cultural background), and behavioral aspects (e.g., health behaviors, learning behaviors, and related outcome expectations). Under these two frameworks, it is not hard to see that among the above factors that can affect oral healthcare practices, psychological attributes (e.g., attitude, confidence) are one of the most modifiable factors at the individual level. Attitude refers to a psychological tendency that is expressed by evaluating a particular entity with some degree of favor or disfavor (31), influenced by individuals’ affection, behavioral tendency, and cognition level (e.g., knowledge/direct experience) (32). While confidence refers to the level of one’s belief in themselves, based on previous experience/evidence, that certain future events will occur as expected (33), influenced by personal expertise, authority, past experience/record, evidence/precedent, identity, belief, and shared group reinforcement (34). Studies (29, 30) indicate that confidence is more likely to be influenced by past success and accomplishments compared to attitude. In summary, attitude and confidence can shape nursing practitioners’/future nursing practitioners’ oral healthcare practices.

Nurses are frontline professionals who play a critical role in managing oral health in clinical, long-term care, and community settings. Their responsibilities include conducting routine oral assessments, providing specialized care for at-risk patients, and delivering essential oral health education (24, 27). Nursing students, who will be future oral care practitioners, are in their vital period of cultivating their attitudes and confidence towards oral healthcare, which may have a substantial impact on their future oral healthcare practices. Additionally, lack of positive attitude and confidence can lead to increased occurrences of missed oral healthcare in practice (35, 36), which may subsequently result in the development of oral diseases (37), social and psychological issues (38), and even severe diseases like cardiovascular disease, diabetes, chronic obstructive pulmonary disease, obstructive sleep apnea (18), Alzheimer’s disease (19) and cancers (20). Multiple studies (39–42) investigated oral healthcare-related attitudes among nursing students, and the levels of these attitudes are generally acceptable globally. For instance, a study (43) from Japan found that nursing students (*N* = 101) showed a medium level of attitudes towards assessing patients’ oral health, scoring 32.50 out of 40, in 2020. Three studies (44–46) conducted in 2012, 2018, and 2020 (*N* = 723/426/1672) in China showed that 53.8% of nursing students expressed interest in oral healthcare, and 65.3 and 79.7% of medical students held positive attitudes towards oral healthcare. Since all the above participants were students during their medical education period, and attitudes stem from knowledge and direct experience, regardless of the quality and quantity of oral healthcare education they received (47, 48), students’ attitudes towards oral healthcare are generally acceptable. However, a review of the literature reveals that the status of confidence in oral healthcare among nursing students is concerning (39–42). For instance, a study (49) conducted in 2016 (*N* = 31) in the United States found that nursing students had a low confidence level, with an average score of 13.13 out of 30 in providing pediatric oral healthcare, and 34% lacked confidence in oral diagnosis of dental caries. Another study (43) from Japan showed that nursing students (*N* = 101) had a confidence score of only 22.09 out of 45 in identifying the categories of oral diseases. Confidence is influenced by previous success and accomplishments (29, 30); therefore, it can be inferred that, with insufficient successful direct experience, as a result of most nursing students having limited opportunity (e.g., limited chance to do clinical practice) to gain substantial successful experiences, this affected their confidence substantially. Lastly, although there are some studies on nursing students’ attitudes and confidence in oral healthcare worldwide, a literature review conducted by the authors of this study found a great paucity of research in this field in China.

Regular measurement of nursing students’ status and associated factors in oral healthcare-related attitudes and confidence can not only prompt a comprehensive understanding of the current status but also provide clues for deploying specific interventions (50). Through a review of the literature, we found that most existing scales are not suitable for measuring nursing students’ attitudes and confidence towards oral healthcare (43, 51, 52) and have the following characteristics: (a) Attitudes and confidence are only sub-dimensions but not the primary focus of measurement. For instance, the Chinese version of the Short Form of Health Literacy Dental scale (51) was introduced into China in 2020 by Yan Wen, which primarily aims to assess oral health literacy among Chinese healthcare professionals. The Oral Health Examination Competency Questionnaire (43), developed by Haresaku in 2019, paid more attention to the oral measurement ability of nursing students, including the ability to read the oral check pictures(e.g., X-ray) and whether an oral referral will be needed; (b) however, it did not possess the necessary validity and reliability outcome indicators. For example, the scale (52) was optimized from the original Prenatal Oral Health Program (53) by Dsouza in 2017 to evaluate nursing students’ knowledge, attitudes, and behaviors regarding oral health services; however, it lacks a comprehensive validation process and validity/reliability outcome indicators. Furthermore, the Dental Coping Beliefs Scale, developed by Wardh in 2005 to evaluate nursing students’ oral health care priorities, also lacks a comprehensive validation process (54). To address these gaps, we found that a scale called the Attitudes and Confidence in Oral Healthcare for Nursing students (ACORN), developed by Jacqueline Rojo (50) among 244 nursing undergraduates in Australia in 2023, specifically designed to measure nursing students’ attitudes and confidence towards oral healthcare. This scale includes two dimensions (attitudes and confidence) and 24 items, with an Item-Level Content Validity Index (I-CVI) of 0.80, a Scale-Level Content Validity Index (S-CVI) of 0.80, an overall Cronbach’s alpha of 0.94, Cronbach’s alpha of the attitude sub-scale of 0.92, and Cronbach’s alpha of the confidence sub-scale of 0.96. All indicators suggest that the ACORN has excellent reliability. It also has strong validity, as indicated by confirmatory factor analysis (CFA), *χ*^2^ = 327.28, df = 215, *p* < 0.01, GFI = 0.83, TLI = 0.95, CFI = 0.96, RMSEA = 0.06, which fully supports the two-dimensional theoretical model.

Therefore, this study aims to adapt the original ACORN scale to Chinese culture to provide a validated measurement tool for evaluating the attitudes and confidence toward oral healthcare among Chinese nursing students, thereby promoting the provision of high-quality oral healthcare in future clinical practice.

## 2 Materials and methods

### 2.1 Study design

A cross-sectional survey was conducted in this study.

### 2.2 Settings

Convenience sampling was used to recruit universities that offer nursing programs for bachelor’s degrees, while cluster and convenience sampling were used to recruit nursing students in these universities. In this study, Chengdu city (Southwest China) and Shenyang city (Northeast China) were chosen based on the geographic locations of the research team members. The data collection was conducted from March 2023 to April 2023. A total of 14 universities offering bachelor’s degree programs in nursing (9 in Chengdu and 5 in Shenyang) were identified through the China Higher Education Student Information Network (CHSI). The research team contacted representatives from 8 of these schools (5 in Chengdu and 3 in Shenyang; more details are in Supplementary Table S1) using a convenience sampling method to explain the study’s purpose, methodology, and contents and to seek their participation and informed consent.

Currently, China’s oral health education system for nurses remains underdeveloped. It is characterized by a lack of standardized curricula, significant regional disparities in teaching materials, and an absence of assessments (55–58). This is compounded by the fact that only 14 institutions nationwide offer specialized oral healthcare programs, with enrollment numbers failing to meet clinical demand (57). Consequently, most dental nurses come from general nursing backgrounds and often exhibit deficits in specialized knowledge and practical skills (55–58). Given the context above and based on a preliminary survey by researchers in this study of the participating universities’ oral health education curricula, this study operationally defines “oral health education” as follows: students must have begun systematic study in both Basic Nursing and Adult Nursing courses, be in their sophomore year or above, and have started oral clinical practicums.

### 2.3 Sample size estimation

The sample size requirement for a cross-sectional survey of the scale is 5–10 times the total number of scale entries (59). In this study, we calculated the sample size by multiplying the number of entries by 10 to 20 times due to both the Exploratory Factor Analysis (EFA) and Confirmatory Factor Analysis (CFA) (60), each of which requires 5 to 10 times the number of total entries, and the total is 10 to 20 times. Additionally, accounting for a 20% attrition rate, the estimated sample size ranged from 288 to 576 participants.

### 2.4 Data collection procedures

Upon receiving approval and informed consent from the representatives of the universities, the researchers tailored data collection plans based on the unique circumstances of each region. In Chengdu, five schools opted for on-site data collection using electronic questionnaires. The research team explained the study’s details to potential participants in classrooms and invited them to scan a QR code to access the e-questionnaire. However, in Shenyang, three to four questionnaire distributors were assigned to each university to handle the one-on-one distribution of the electronic questionnaires to nursing students; data collection in all three Shenyang schools was conducted online. The procedure is as follows: the distributors added potential participants on WeChat/QICQ apps, explained the study’s details, and shared the QR code with eligible students to complete the questionnaire. Students were required to scan the QR code to access the e-questionnaire, which also included an informed consent form. The distributors regularly updated the research team via WeChat to ensure data authenticity.

The questionnaires were hosted on WenJuan Xing,^1^ a widely used online survey platform in China. The platform automatically recorded and stored the data. Each e-questionnaire includes an informed consent form, a separate guidance page explaining the study’s purpose, completion instructions, and estimated time required. The survey officially began only after participants indicated their informed consent. To ensure data integrity, the electronic survey was designed to be submitted only after 100% completion, and each device can submit only once. Only questionnaires completed within a reasonable time frame (at least 120 s) were considered valid. Invalid responses, such as those completed too quickly, those that selected the same option throughout, or those that followed a predictable pattern (e.g., “11111” or “12345”), were excluded from the final dataset.

### 2.5 Inclusion and exclusion criteria for the participants and their questionnaires

**Inclusion criteria:** (a) full-time nursing students (studying their bachelor’s degree in these universities) who have received oral healthcare education before; (b) ability to read and complete the questionnaire; (c) informed consent provided for voluntary participation.

**Exclusion criteria:** (a) questionnaires completed in less than 120 s; (b) responses that show a consistent pattern (e.g., selecting the same option repeatedly or following a predictable sequence like 11111 or 12345).

### 2.6 Instruments

#### 2.6.1 Social demographic information

This questionnaire enquires participants’ social demographic information, including participants’ gender, age, school, and grade, and was designed by the research team after a review of the literature (43, 50, 52, 61–67).

#### 2.6.2 The Attitudes and Confidence in Oral Healthcare for Nursing students (ACORN) scale

The ACORN scale, developed by Jacqueline Rojo in 2023 (50) consists of two dimensions, attitude and confidence, covering a total of 24 items. Each item was rated on a 7-point Likert scale ranging from 1 (strongly disagree) to 7 (strongly agree). The scale demonstrated favorable validity and reliability with an Item-Level Content Validity Index (I-CVI) > 0.78, a Scale-Level Content Validity Index (S-CVI) = 0.8, an overall Cronbach’s alpha of 0.94, the attitude subscale’s Cronbach’s alpha of 0.92, and the confidence subscale’s Cronbach’s alpha of 0.96. EFA extracted two factors (attitude and confidence), and CFA yielded *χ*2 of 327.28, df of 215, *p* < 0.01, GFI of 0.83, TLI of 0.95, CFI of 0.96, and RMSEA of 0.06 (68).

### 2.7 Translation and cultural adaptation procedures

After contacting the original scale author via email, the researchers obtained authorization to translate/introduce the original English version of the ACORN scale into Chinese culture on October 3, 2023, as illustrated in Supplementary Figure S1. The translation and cultural adaptation procedure in this study totally followed the translation, adaptation, and validation of instruments or scales for use in cross-cultural health care research: a clear and user-friendly guideline, including forward translation, back translation, and expert consultation (consultation of the differences between the original English version and the synthesized back-translated version of the ACORN scale with the original author, pilot testing, expert consultation, and bilingual testing) (69).

#### 2.7.1 Forward translation

Two master’s subjects in nursing (LZ) and Chinese language and literature (QG) independently; both were proficient in English and translated the ACORN scale from English to Chinese independently. A third nursing master (XXX), who is also proficient in English, along with the researcher (CX), compared the two translated drafts with the original version. After discussion among the researchers (CX, YL, LL) and three translators, a consensus was reached, and a synthesized Chinese version of ACORN was formed (XXX, CX); more details are in Table S2.

#### 2.7.2 Back translation

Two master’s subjects in nursing (XLF) and Chinese language and literature (YLY) separately, both were proficient in English but had no prior knowledge of the original scale. They independently translated the synthesized Chinese version of ACORN into English. An expert committee composed of the researcher (CX), scale construction expert (YL, LL), and all translators (QG, LZ, XXX, YLY, and XLF) compared the two back-translated drafts. They discussed differences between the back-translated version and the original scale. After a consensus was reached, the synthesized back-translated version of the ACORN (CX) scale was finalized. More details are listed in Table S3.

#### 2.7.3 Expert consultation (cultural adaptation)

##### 2.7.3.1 Consultation of the differences between the original English version and the synthesized back-translated version of the ACORN scale with the original author

The synthesized back-translated ACORN (CX) scale was sent to the original English version author (Jacqueline Rojo) via email to clarify ambiguities discussed by the expert committee and differences between the original English version and the synthesized back-translated version of the ACORN scale. After adjustments, the Chinese version of the ACORN (CX) scale was developed, with additional details shown in Supplementary Figure S2 and Supplementary Table S4.

##### 2.7.3.2 Pilot testing

Methodological literature outlines a standard procedure for scale translation and cross-cultural adaptation, which includes forward translation, synthesis of the forward translations, back translation, synthesis of the back translations, pilot testing, bilingual testing, and formal research (69). Pilot testing involves a preliminary study with 10–40 participants from the target population to assess the clarity and comprehensibility of the scale’s instructions, items, and response format (69). Additionally, the engagement of an expert consultation throughout this process is highly recommended to evaluate and ensure the conceptual and content equivalence of all items (69). Twenty nursing students who met the inclusion/exclusion criteria of the formal survey and provided informed consent were invited through convenience sampling to participate in pilot testing. We assessed whether each item in the scale was clear and easy to understand by using the adjusted Chinese version of ACORN, along with two options, “YES” or “NO” for response selection, and an extra blank line for feedback on whether there will be any other suggestions, at the end of each item. Items would be revised unless 100% of respondents selected “Yes”. Based on this criterion, the results indicated that students understood most items well. While five items fell below the 100% threshold and were revised, the remaining 19 out of 24 items achieved a perfect understanding rate. Additionally, based on their suggestions, we added explanations for certain terminologies to the ACORN (CX) scale to improve readability, including dental referrals, plaque, tartar, receding gums, and worn-down teeth. Demographic information for the pilot testing participants is available in Supplementary Table S5. The items of the adjusted Chinese version ACORN, along with participants’ corresponding ‘Yes’ response rates, participants’ feedback, and our subsequent revisions, are provided in Supplementary Table S6.

All 20 participants, who were from the eight universities included in this study and who met the identical inclusion and exclusion criteria used in both the pilot and formal studies, voluntarily continued the formal survey.

##### 2.7.3.3 Expert consultation

Ten experts were invited for the Delphi method (70) (including six experts from clinical nursing, three from nursing education, and one from nursing research). They were invited to assess the importance of items, the relevance of content, and the clarity of language expression. The Chinese version of the ACORN scale was further revised based on all this feedback. Some explanations for certain terminologies were added to the ACORN (CX) scale-pressure area care, healthy teeth, and oral pain, as well as some other revisions were made, including added word “recommend” before “dental referrals” and changed “lip ulcers” to “oral ulcer.” More details, including experts’ demographic information and the revised scale, are presented in Supplementary Tables S7, S8.

##### 2.7.3.4 Bilingual testing

To establish standard equivalence and further validate the conceptual, semantic, content, and structural equivalence of a scale, a bilingual test was conducted (69). This involves a pilot study with a minimum of five bilingual participants (69). Bilingual testing derives its value from the unique bicultural perspective of bilingual individuals, who act as “conceptual proofreaders” and “cultural detectors” (69). Their direct comparison of language versions reveals deep-seated issues-such as conceptual nuances, cultural misalignments, and awkward idioms-that professional translation and monolingual testing typically miss. This is critical for establishing true conceptual and cultural equivalence, thus ensuring the validity and comparability of cross-cultural research. Ten nursing students who met the inclusion criteria of this study and were proficient in English (there was no overlap between participants in the pilot testing and the bilingual testing) were invited to complete a questionnaire comprising both the original English ACORN scale and a randomly shuffled Chinese version of the ACORN scale to measure the equivalence between the two scales. Participants in the bilingual testing voluntarily participated in the subsequent formal survey because they met the inclusion and exclusion criteria of this study and were also from the eight universities included in this study. Demographic information of participants, the questionnaire applied, and statistical results from the bilingual assessment are shown in Supplementary Tables S9–S11, respectively.

### 2.8 Ethical consideration

This study was approved by the Biomedical Ethics Committee of West China Hospital, Sichuan University (Reference Number: 2024–279). This study received data collection permissions from all included universities. Informed consent was obtained from all participants in this study. All procedures conducted in this study were in accordance with the Declaration of Helsinki (71).

### 2.9 Data analysis methods

#### 2.9.1 Item analysis

This study employs both the critical ratio (CR) method and the coefficient correlation method to assess the suitability and reliability of scale items. In the CR method, the total scale scores are ordered from lowest to highest, with the lowest 27% forming the low-score group and the highest 27% forming the high-score group. A *t*-test was performed on the same item for the high- and low-score groups. If the difference is not statistically significant (*p* > 0.05, the tests involving *p*-values throughout this study were all two-tailed) or the CR is less than 3.00, the item was removed. In the correlation coefficient method, which examines the correlation between each item’s score and the overall scale score. Items with a correlation coefficient of r ≥ 0.40 (*p* < 0.05) were retained.

#### 2.9.2 Content validity

In this study, ten experts were involved in the cultural adaptation process, assessing the content validity of the scale. Experts were requested to rate each item’s relevance to oral healthcare attitudes and confidence by using a Likert scale ranging from “not important” to “very important” (score from 1 to 4). The I-CVI and the S-CVI were calculated. Both of the I-CVI and S-CVI ≥ 0.80 will be taken as good content validity of the scale (72).

#### 2.9.3 Structural validity—EFA

This study employed both EFA and CFA to assess the structural validity of the scale. Employing the random number table method in SPSS 26.0, we split the data into two independent, equal-sized subsamples (EFA: *N* = 268; CFA: *N* = 268). This rigorous partitioning ensures that the cases in each set are mutually exclusive, thereby eliminating information leakage and upholding the validity of the statistical tests derived from the CFA.

Before conducting the EFA, the suitability of the data for analysis was evaluated using the Kaiser–Meyer–Olkin (KMO) measure and Bartlett’s test of sphericity. EFA was deemed appropriate if the KMO value exceeded 0.50 and Bartlett’s test produced a *p*-value less than 0.05 (73). The EFA was conducted using principal component analysis and varimax orthogonal rotation, extracting common factors with eigenvalues of at least 1.000. Structural validity was considered strong if item factor loadings on their respective factors exceeded 0.50, no items showed multiple loadings, and the cumulative variance contribution of the scale was above 50% (74).

#### 2.9.4 Reliability check

Internal consistency reliability was used to assess the internal consistency of each domain and the whole scale. The test–retest reliability coefficient was used to examine the stability of the scale.

##### 2.9.4.1 Cronbach’s alpha coefficient

Internal consistency refers to the correlation between items, and a high correlation among items in the same dimension indicates high internal consistency (75). It is evaluated by calculating Cronbach’s alpha coefficient of the dimension/whole scale. If Cronbach’s alpha coefficient is >0.70, it means good internal consistency reliability of the scale (76).

##### 2.9.4.2 Test–retest reliability

Test–retest reliability refers to administering the same measurement tool to the same group at different time points to evaluate the consistency and stability of the results/scale (77). In this study, we tested test–retest reliability using the same scale within the same subjects after a 2-week interval. A random sampling of 20 nursing students in one of the universities in Chengdu was re-surveyed to assess the scale’s test–retest reliability. The correlation coefficient of the scores obtained from these two surveys was calculated by the Pearson product–moment correlation coefficient which ranges from 0 to 1, and the higher of the results indicates the better consistency between two tests, as well as good stability of the scale. The test–retest reliability coefficient value greater than 0.70 will be considered satisfactory and excellent (78).

#### 2.9.5 Structural validity—CFA

Following EFA, CFA was performed to validate the scale’s structure. A good model fit was indicated if the chi-square/degree of freedom ratio (χ^2^/df) was less than 5, the CFI and IFI were 0.90 or higher (79), and the RMSEA was below 0.08 (80).

### 2.10 Statistical analysis

In this study, SPSS 26.0 and AMOS 26.0 were applied for statistical analysis. Descriptive statistical analysis was applied by using mean ± standard deviation (x̄ ± s), frequency, and percentages (%) to present the results. In item analysis, we employed the CR method and the correlation coefficient method, and content validity and structural validity were assessed. The reliability test involved internal consistency and the test–retest reliability coefficient method. Differences were considered statistically significant if *p* < 0.05.

## 3 Results

This study included eight universities: five from Chengdu and three from Shenyang. A total of 550 questionnaires were distributed, except 14 questionnaires were excluded as their completion was within 120 s. Five hundred thirty-six questionnaires were included with a retrieval rate of 97.45%, of which 298 participants (55.60%) were from Chengdu, and 238 (44.40%) were from Shenyang. Participants’ age ranged from 19 to 24 years, with a mean age of (21.17 ± 1.16) years. Most participants were female (*N* = 389, 72.57%), comprising sophomores (25.00%), juniors (34.51%), and seniors (40.49%). More details are presented in Supplementary Table S12.

### 3.1 Item analysis

Item analysis was conducted to screen for potentially irrelevant items. Using the CR method, a significant difference was found between the top 27% of scorers (156.80 ± 5.81; min total score = 149, max total score = 168) and the bottom 27% (105.88 ± 15.37; min = 24, max = 123). All items demonstrated high discriminant validity, with CR values ranging from 13.12 to 23.14. An independent-samples *t*-test confirmed that the score differences between these groups were statistically significant (*p* < 0.05). Furthermore, all items showed strong positive correlations with the total scale score (*r* = 0.56 to 0.78, *p* < 0.05), indicating excellent homogeneity for both the entire scale and each item. Based on these robust results, all items were retained. Detailed results are presented in Supplementary Table S13.

### 3.2 Content validity-expert evaluation

The Item-Level Content Validity Index of 24 items ranges from 0.80 to 1.00, and the Scale-Level Content Validity Index was 0.94, indicating a favorable content validity of the Chinese version (72, 81). More details, including the demographics of the expert involved, are in Supplementary Tables S7,S8.

### 3.3 Exploratory factor analysis (EFA)

The results indicate that the Kaiser–Meyer–Olkin (KMO) index was 0.954, and Bartlett’s test of sphericity showed statistical significance (*p* < 0.001), confirming the applicability of factor analysis (74). The results of EFA demonstrated that the optimal factor structure comprises three factors with a total of 22 items and a cumulative variance contribution rate of 66.33%. The first factor, consisting of 8 items (Q1-Q8), explains 25.10% of the variance; the second factor, with four items (Q9-Q12), explains 12.62% of the variance; and the third factor, including 10 items (Q13-Q22), explains 28.61% of the variance. The items in each dimension are shown in Table 1.

**Table 1:** Cronbach’s alpha coefficients for the Chinese version of the ACORN scale.

Items		Cronbach’s alpha
Factor 1: attitudes toward oral care		0.92
Q1	It is a nursing responsibility to ensure patients have good oral hygiene.	
Q2	Providing patients’ oral care is as important as pressure area care, such as preventing pressure injuries.	
Q3	It is important for nurses to actively recommend dental referrals for patients when necessary. (Based on the patient’s condition, you should recommend patients under your care to dental services for treatment.)	
Q4	Nurses are well-positioned to detect oral health problems early.	
Q5	Providing oral care is always required for patients with dysphagia.	
Q6	It is the nurses’ duty to remind patients of their oral health care.	
Q7	Nurses should have the skills to conduct oral health assessments.	
Q8	It is important to ensure that patients of all ages receive adequate oral care.	
Factor 2: confidence in oral care practices		0.85
Q9	Discussing oral health with patients.	
Q10	Discussing with patients the relationship between oral health and their overall health status or potential health issues.	
Q11	Providing comprehensive oral care for patients who are conscious but unable to get out of bed.	
Q12	Recommending referring patients to dental services after your oral health assessment.	
Factor 3: confidence in diagnosing oral diseases		0.94
Q13	Dental caries	
Q14	Oral pain: pain in the mouth caused by various reasons.	
Q15	Oral ulcers	
Q16	Food granule	
Q17	Dental plaque: a microbial community that adheres to the surface of teeth or other soft tissues in the mouth.	
Q18	Dental calculus/Tartar: tartar is formed by the mineralization of plaque and other deposits on the tooth surface.	
Q19	Receding gums: the gradual retreat of the gum margin towards the root apex.	
Q20	Inflamed gums (e.g., swollen gums)	
Q21	Broken teeth	
Q22	Tooth wear: a condition characterized by the progressive loss of hard dental tissues, primarily caused by mechanical friction.	
Overall score		0.95

### 3.4 Confirmatory factor analysis (CFA)

**Figure 1:**
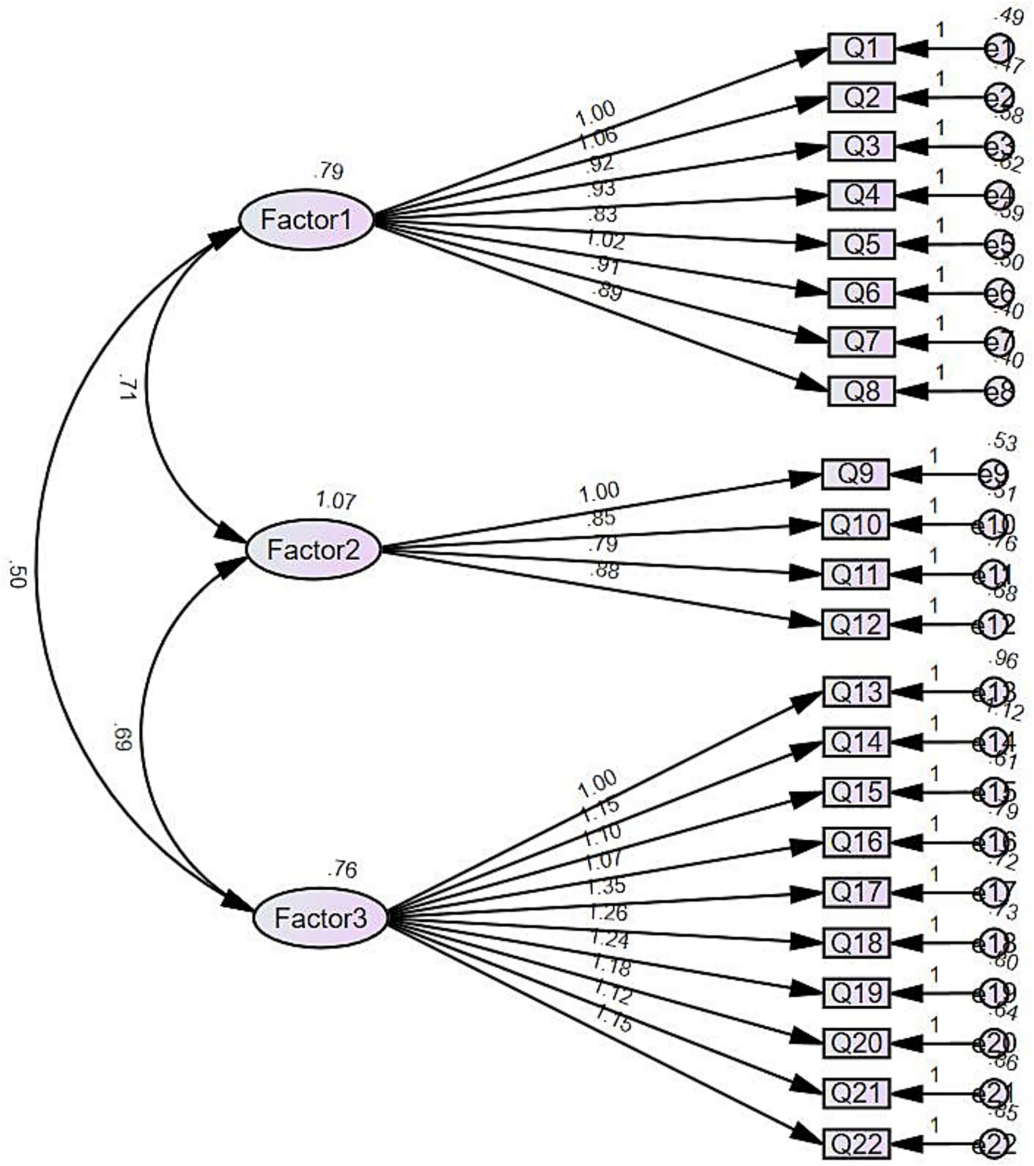
Path diagram from CFA.

The CFA results demonstrated a satisfactory model fit (82), with *χ*2/df = 1.97, GFI = 0.88, CFI = 0.95, NFI = 0.90, TLI = 0.94, and RMSEA = 0.06. Additionally, the model structure has standardized regression weights ranging from 0.67 to 0.82, as depicted in Figure 1.

### 3.5 Reliability

The Cronbach’s alpha coefficient of the whole Chinese version scale was 0.95, with 0.92 for the attitude subscale, 0.85 for confidence in performing, and 0.94 for confidence in identifying, which were all excellent (83), as presented in Table 1. Additionally, the test–retest reliability of the Chinese version scale was satisfactory and excellent (78), as indicated by an intraclass correlation coefficient value of 0.76.

## 4 Discussion

Driven by international advocacy, recently, many countries, including the United States, Turkey, and China, are progressively integrating oral health education into undergraduate nursing curricula (41, 42, 84–86). However, due to the delayed recognition of the importance of oral healthcare, there is a substantial lack of awareness among nursing students, affecting their attitudes and confidence in oral healthcare. Additionally, upon a review of the literature (43, 51, 52), we found that, currently, there is no available, not even to say validated scale, to help in measurement of Chinese nursing students’ attitude and confidence related to oral healthcare. The Attitude and Confidence with Oral Healthcare among Nursing Students (ACORN) scale, with moderate item quantity and operational feasibility, enables dynamic and real-time measurements of Chinese nursing students’ attitude and confidence related to oral healthcare, which can provide related stakeholders with insights into students’ status of their attitude/confidence related to oral healthcare, and also empower nursing students themselves to shape their future practices in oral healthcare. Thus, in this study, we introduced and tested the psychometric properties of this novel and practical attitude and confidence measurement scale for nursing students in Chinese culture.

In this study, a total of 8 universities in Chengdu city and Shenyang city were surveyed separately, with 536 effective questionnaires (97.5%) retrieved. The sample size in this study was not only sufficient for structural validity checking, for both the Exploratory factor analysis (EFA) and Confirmatory Factor Analysis (CFA), comparatively speaking, it had twice sampled as much as in the original English version (*N* = 244) and a higher retrieval rate than the original English version study (94.20%) (50), which makes the results from this study more robust and trustful. Similarly with the original English version study, most nursing students in this study were also female (85.30%, in this study, the proportion was 72.57%), and young students (mean age was 27.83 and 28.67 in group 1 and group 2, separately, in the original English version, while 21.17 ± 1.16 years in this study).

According to the Knowledge, Attitude/Belief, Practice (KAP) theory, human behavior change encompasses three sequential processes: acquiring knowledge, forming beliefs and attitudes, and shaping behavior (28). While the Social Cognitive Theory (SCT) emphasizes that an individual’s actions are the result of multiple interacting factors, including personal, environmental, and behavioral factors (29, 30). It is evident that personal factors, particularly psychological attributes, play a critical role in the formation and modification of behavior and are the easiest to change. Among these personal factors, attitudes and confidence are especially important, as they directly influence nursing students’ behavior and actions according to the theories mentioned above, and even their future oral healthcare practices. This study suggests that there may be a substantial link between a person’s attitude, self-confidence, and their resulting behavior and actions. However, a literature review conducted by the authors of this study indicates a lack of a theoretical framework explaining how an individual’s attitude and self-confidence contribute to their behavior and ultimately lead to action. We can make a bold assumption that attitude and self-confidence may directly influence behavior, which in turn leads to specific actions.

In this study, 536 questionnaires were randomly divided into two equal groups using SPSS’s random number table function. On the one hand, one group underwent an EFA to investigate the structure of the 22 items across the three dimensions of the Chinese version of ACORN (attitudes toward oral care, confidence in oral care practices, and confidence in diagnosing oral diseases). The EFA revealed that the three factors of the ACORN scale accounted for a cumulative variance contribution rate of 66.33%, indicating a strong contribution. There is a substantial distinction between the original English version scale with the Chinese version scale is, the split of the previously “confidence” dimension in the original English version, into “confidence in oral care practices,” and “confidence in diagnosing oral diseases” dimensions in the Chinese version ACORN. In the original scale, there are only two dimensions, attitude and confidence, with the confidence dimension treated as a single overarching category. However, the scale’s format separates the confidence dimension into two modules, “confidence in oral care practices” and “confidence in diagnosing oral diseases.” After cultural adaptation into China, this division into modules” confidence in oral care practices” and “confidence in diagnosing oral diseases,” was maintained in the Chinese context, which substantially enhances the understanding of confidence and its practical categorization and application, emphasizing the distinct aspects of confidence demonstrated by nursing students in oral healthcare practice. A more detailed classification helps enriching the details of the Chinese version ACORN scale, and authors believe that this classification can help clarify the sources of nursing students’ confidence when facing clinical tasks. Additionally, it may also be related to the fact that nursing students have less knowledge/practice/experience about the diagnosis of oral diseases (87), leading to the large difference between these two dimensions. On the other hand, the second group was used for CFA, which demonstrated a good model fit. In summary, the Chinese version of ACORN demonstrated excellent structural validity.

Also, the reliability performance of the Chinese version of ACORN is excellent. In this study, the Item-Level Content Validity Index ranges from 0.80 ~ 1.00 and the value of Scale-Level Content Validity Index was 0.94, both were greater than 0.80 (72), indicating the authenticity and accuracy of the content expressed in the Chinese version of the ACORN scale. Additionally, this study utilized Cronbach’s alpha coefficient to evaluate the internal consistency reliability of the Chinese version of the ACORN scale (75). The Cronbach’s alpha coefficients for the overall scale was 0.95, and for each dimension were 0.92, 0.85, and 0.94separately, which all greater than 0.70 (76), demonstrating a good internal consistency of the Chinese ACORN scale. Furthermore, the test–retest reliability coefficient was 0.76, which is greater than 0.70 (78) and reflects the scale’s good stability.

Convenience sampling was used in this study to include nursing universities that offer a bachelor’s degree in nursing; however, these universities were located only in the northeast (Shenyang city) and southwest (Chengdu city) regions of China, which may have led to an inadequate representation of the sample. Future research will be needed to validate and assess the psychological properties of the Chinese version of the ACORN scale in other regions of China before its widespread use in this country.

## 5 Conclusion

The Chinese version of the ACORN scale was translated, back-translated, and culturally adapted in strict accordance with Brislin’s translation process. The Chinese version scale has demonstrated excellent reliability and validity, serving as a specific and reliable tool for measuring nursing students’ attitudes and confidence in oral healthcare. However, it is important to acknowledge that this study’s reliance on convenience and cluster sampling may have introduced selection bias, reduced the statistical independence of responses within clusters, and limited the generalizability of the findings. Future measurements among Chinese nursing students can guide the development of interventions to improve nursing students’ attitudes and confidence in oral healthcare, ultimately contributing to the provision of high-quality oral healthcare services by future nurses.

## Data Availability

The original contributions presented in the study are included in the article/Supplementary material, further inquiries can be directed to the corresponding authors.

## Footnotes

1
https://www.wjx.cn/
