# Supplementary material for: Transcultural adaptation and psychometric evaluation of the Chinese version of attitude and confidence with oral healthcare among nursing students scale: a cross-sectional survey in China
Source: Front Public Health. 2025 Nov 25;13:1689136. doi: 10.3389/fpubh.2025.1689136 (PMC12685846; doi:10.3389/fpubh.2025.1689136)
Supplement: Supplementary file 1 [file Data_Sheet_1.pdf]

Supplementary Materials

1. Figures

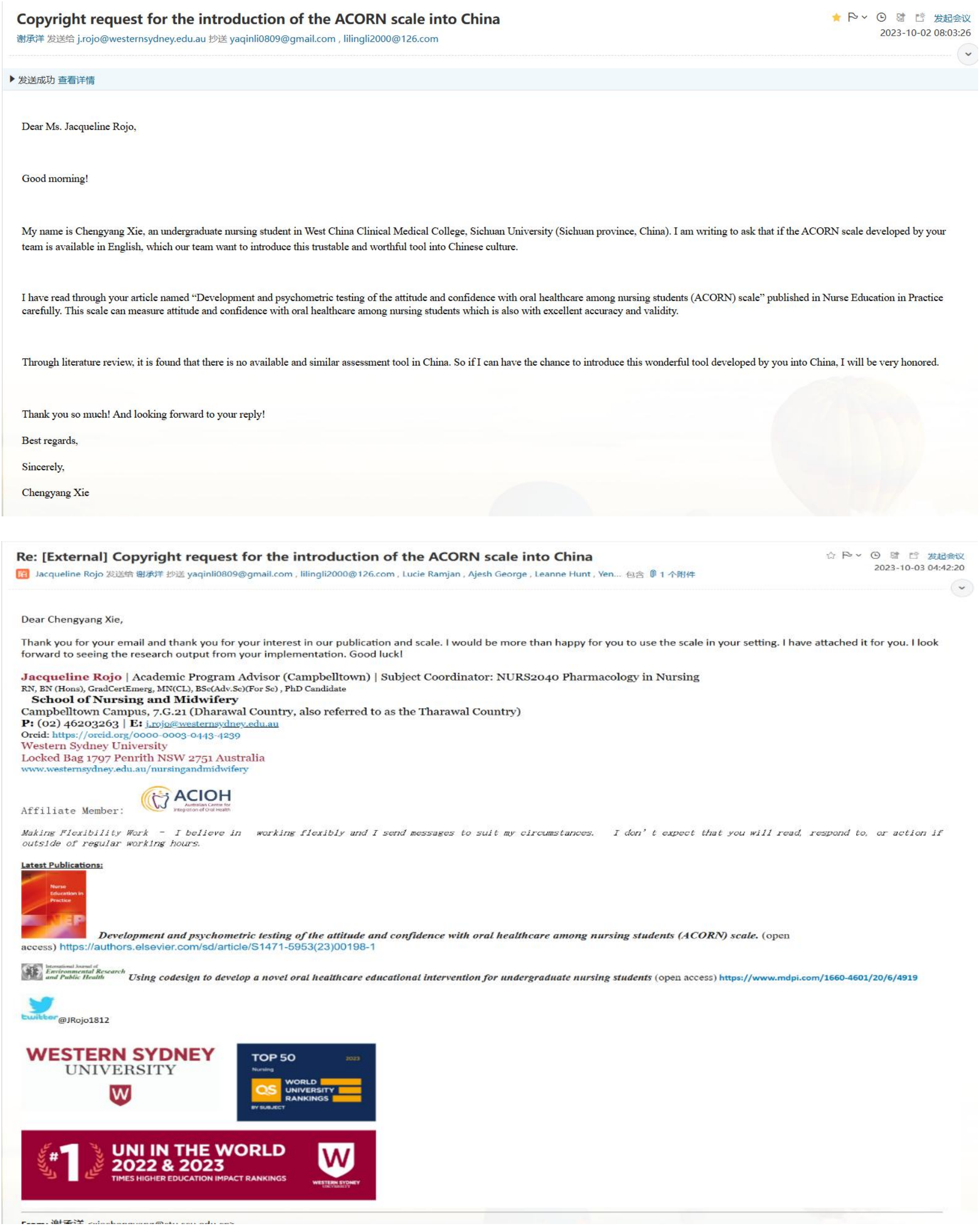

Copyright request for the introduction of the ACORN scale into China

谢承洋 发送给 j.rojo@westernsydney.edu.au 抄送 yaqinli0809@gmail.com , lilingli2000@126.com

2023-10-02 08:03:26

发送成功 查看详情

Dear Ms. Jacqueline Rojo,

Good morning!

My name is Chengyang Xie, an undergraduate nursing student in West China Clinical Medical College, Sichuan University (Sichuan province, China). I am writing to ask that if the ACORN scale developed by your team is available in English, which our team want to introduce this trustable and worthful tool into Chinese culture.

I have read through your article named "Development and psychometric testing of the attitude and confidence with oral healthcare among nursing students (ACORN) scale" published in Nurse Education in Practice carefully. This scale can measure attitude and confidence with oral healthcare among nursing students which is also with excellent accuracy and validity.

Through literature review, it is found that there is no available and similar assessment tool in China. So if I can have the chance to introduce this wonderful tool developed by you into China, I will be very honored.

Thank you so much! And looking forward to your reply!

Best regards,

Sincerely,

Chengyang Xie

Re: [External] Copyright request for the introduction of the ACORN scale into China

Jacqueline Rojo 发送给 谢承洋 抄送 yaqinli0809@gmail.com , lilingli2000@126.com , Lucie Ramjan , Ajesh George , Leanne Hunt , Yen... 包含 1 个附件

2023-10-03 04:42:20

Dear Chengyang Xie,

Thank you for your email and thank you for your interest in our publication and scale. I would be more than happy for you to use the scale in your setting. I have attached it for you. I look forward to seeing the research output from your implementation. Good luck!

Jacqueline Rojo | Academic Program Advisor (Campbelltown) | Subject Coordinator: NURS2040 Pharmacology in Nursing
RN, BN (Hons), GradCertEmerg, MN(CL), BSc(Adv.Sc)(For Sc) , PhD Candidate
School of Nursing and Midwifery
Campbelltown Campus, 7.G.21 (Dharawal Country, also referred to as the Tharawal Country)
P: (02) 46203263 | **E:** j.rojo@westernsydney.edu.au
Orcid: https://orcid.org/0000-0003-0443-4239
Western Sydney University
Locked Bag 1797 Penrith NSW 2751 Australia
www.westernsydney.edu.au/nursingandmidwifery

Affiliate Member:

Making Flexibility Work – I believe in working flexibly and I send messages to suit my circumstances. I don't expect that you will read, respond to, or action if outside of regular working hours.

Latest Publications:

Development and psychometric testing of the attitude and confidence with oral healthcare among nursing students (ACORN) scale. (open access) https://authors.elsevier.com/sd/article/S1471-5953(23)00198-1

Using codesign to develop a novel oral healthcare educational intervention for undergraduate nursing students (open access) https://www.mdpi.com/1660-4601/20/6/4919

@JRojo1812

Fig S1. Emails for authorization

Request for expertise on translated scale

谢承洋 发送给 j.rojo@westernsydney.edu.au 抄送 yaqinli0809@gmail.com , lilingli2000@126.com 包含 1 个附件

2023-10-16 08:00:37

发起会议

▸ 发送成功 查看详情

Dear Ms. Jacqueline Rojo,

Good morning! I am Chengyang Xie. I am writing to ask if you could give some expertise to compare the differences between the original ACORN scale and the current scale which has been forward-translated and back-translated in order, and now it' s a back-translated version in English. I have attached this version to this email for your convenience.

As part of the translation process, I have completed the forward translation and the blind back-translation of the questionnaire to ensure its accuracy and cultural appropriateness. However, according to the methodology, which needs the original author to compare the original scale with this back-translated scale to evaluate the similarity of the instructions items in wording, sentence structure and meaning of both versions to ensure the translation quality, if it is allowed by your attention and time. I believe that your insights and feedback, as the original author, would be valuable in making sure that the translated questionnaire maintains the intended meaning and context. Your feedback will be treated with the utmost confidentiality, and we will acknowledge your contribution appropriately in the final publication if it is good for you.

If you have any questions or concerns about the translation, please feel free to contact me. Thank you so much! And looking forward to your reply!

Best regards,

Sincerely,

Chengyang Xie

普通附件 (1 个)

ACORN-BTL 20231015.pdf (73.38K)

下载 预览 保存到个人网盘

Re: [External] Request for expertise on translated scale

Jacqueline Rojo 发送给 谢承洋 抄送 yaqinli0809@gmail.com , lilingli2000@126.com

2023-10-23 09:06:31

发起会议

Hi Chengyang,

I hope this email finds you well and I apologise for the delay in getting back to you. We have reviewed your translation and unfortunately we will not be able to endorse it as the translation deviates from the original meaning of the scale for numerous items. One example of this is the use of the word "Diffident" in place of "Not confident".

Jacqueline Rojo | Academic Program Advisor (Campbelltown) | Subject Coordinator: NURS2040 Pharmacology in Nursing
RN, BN (Hons), GradCertEmerg, MN(CL), BSc(Adv.Sc)(For Sc) , PhD Candidate
School of Nursing and Midwifery
Campbelltown Campus, 7.G.21 (Dharawal Country, also referred to as the Tharawal Country)
P: (02) 46203263 | **E:** j.rojo@westernsydney.edu.au
Orcid: https://orcid.org/0000-0003-0443-4239
Western Sydney University
Locked Bag 1797 Penrith NSW 2751 Australia
www.westernsydney.edu.au/nursingandmidwifery

Affiliate Member: ACIOH

Making Flexibility Work – I believe in working flexibly and I send messages to suit my circumstances. I don' t expect that you will read, respond to, or action if outside of regular working hours.

Latest Publications:

Development and psychometric testing of the attitude and confidence with oral healthcare among nursing students (ACORN) scale. (open access) https://authors.elsevier.com/sd/article/S1471-5953(23)00198-1

Using codesign to develop a novel oral healthcare educational intervention for undergraduate nursing students (open access) https://www.mdpi.com/1660-4601/20/6/4919

twitter @JRojo1812

WESTERN SYDNEY UNIVERSITY

TOP 50 WORLD UNIVERSITY RANKINGS

#1 UNI IN THE WORLD 2022 & 2023

Item	Translation
1	It is nursing staffs' responsibility to ensure that patients maintain good oral hygiene
2	Providing patients' oral care is as important as pressure area care.
3	It is important for nurses to actively make referrals for dental patients when necessary
4	Nurses are ~~have the ability~~ in a good position ~~of~~ to detect ~~early detection of~~ oral health problems early.
5	Providing oral care is always required for patients with dysphagia
6	It is nurses' duty to remind patients of their oral health care
7	Nurses should have the skills to conduct oral health assessments.
8	It is important to ensure that patients of all ages receive adequate oral care.
1	Discussing oral health with patients.
2	Discussing with patients the relationship between oral health and their health problem ~~status~~
3	Providing comprehensive oral care for patients who are conscious but unable to get out of bed.
4	Referring patients to dental services after your oral health assessment.
1	Healthy gums/gingiva
2	Healthy teeth
3	Dental caries
4	Oral pain
5	Lip ulcers
6	Food granule
7	Dental plaque
8	Dental calculus
9	Receding gums
10	Swollen gums
11	Inflamed gums ~~Cracked tooth~~
12	Tooth wear

Figure S2. Request the original author to compare the differences between the back-translated composite version of the scale and the original version of the scale

2. Tables

Table S1. Universities included in this study

University Name	Location
Sichuan University	Chengdu, Sichuan Province
University of Electronic Science and Technology of China	Chengdu, Sichuan Province
Chengdu University of Traditional Chinese Medicine	Chengdu, Sichuan Province
Chengdu University	Chengdu, Sichuan Province
Chengdu Medical College	Chengdu, Sichuan Province
China Medical University	Liaoning, Shenyang Province
Liaoning University of Traditional Chinese Medicine	Liaoning, Shenyang Province
Shenyang Medical College	Liaoning, Shenyang Province

Table S2. Forward translation-the synthesized Chinese version ACORN scale

Item	Revisions on items after discussion	The Synthesized Chinese version ACORN scale
Q1	1. Defined the translation format for patients.<br>2. Changed term "nursing staff" to "nurses" .	确保患者保持良好的口腔卫生是护士的职责。
Q2	1. Defined the translation format for patients.<br>2. Uniformed the expression of "pressure area care".	患者的口腔护理与提供受压部位皮肤护理一样重要。
Q3	Changed term "nursing staff" to "nurses" .	对于护士而言，在必要时启动牙科转诊非常重要。
Q4	Changed term "nursing staff" to "nurses" .	护士有能力早期发现口腔健康问题。
Q5	Defined the translation format for patients.	对于吞咽困难的患者始终需要提供口腔护理。
Q6	1. Defined the translation format for patients.<br>2. Changed term "nursing staff" to "nurses" .	提醒患者注意口腔保健是护士的职责。
Q7	Changed term "nursing staff" to "nurses" .	护士应掌握进行口腔健康评估的技能。
Q8	Defined the translation format for patients.	确保所有年龄段的患者获得足够的口腔护理非常重要。
Q9	Defined the translation format for patients.	与患者讨论口腔健康问题。
Q10	Defined the translation format for patients.	与患者讨论口腔健康与自身健康状况之间的关系。
Q11	Defined the translation format for patients.	为意识清醒但无法下床的患者提供全面的口腔护理。
Q12	Defined the translation format for patients.	在您进行口腔健康评估后，必要时将患者转诊至口腔诊疗机构。
Q13	/	健康的牙龈
Q14	/	健康的牙齿
Q15	/	龋齿
Q16	/	口腔疼痛

Q17	/	唇部溃疡
Q18	/	食物残渣
Q19	/	牙菌斑
Q20	/	牙石（牙结石）/牙垢
Q21	/	牙龈萎缩
Q22	/	牙龈肿胀
Q23	Changed "tooth fracture" to "cracked tooth".	断牙
Q24	/	牙齿磨损

Table S3. Back translation-the synthesized back-translated version ACORN scale

Item	Revisions on items after discussion	The Synthesized Chinese version ACORN scale
Q1	/	It is nursing staffs' responsibility to ensure that patients maintain good oral hygiene.
Q2	/	Providing patients' oral care is as important as pressure area care.
Q3	/	It is important for nurses to actively make referrals for dental patients when necessary
Q4	/	Nurses have the ability of early detection of oral health problems.
Q5	/	Providing oral care is always required for patients with dysphagia.
Q6	/	It is nurses' duty to remind patients of their oral health care.
Q7	/	Nurses should have the skills to conduct oral health assessments.
Q8	/	It is important to ensure that patients of all ages receive adequate oral care.
Q9	/	Discussing oral health with patients.
Q10	/	Discussing with patients the relationship between oral health and their health status.
Q11	/	Providing comprehensive oral care for patients who are conscious but unable to get out of bed.
Q12	/	Referring patients to dental services after your oral health assessment.
Q13	/	Healthy gums/gingiva
Q14	/	Healthy teeth
Q15	Selected "Dental caries" rather than "Tooth decay" as the translation form.	Dental caires
Q16	/	Oral pain
Q17	/	Lip ulcers
Q18	Selected "Food granule" rather than "Food debris" as the translation form.	Food granule

Q19	/	Dental plaque
Q20	/	Dental calculus
Q21	Selected "Receding gums" rather than "Gum recession" as the translation form.	Receding gums
Q22	Selected "Swollen gums" rather than "Gum swelling" as the translation form.	Swollen gums
Q23	Selected "Cracked tooth" rather than "Tooth fracture" as the translation form.	Cracked tooth
Q24	Selected "Tooth wear" rather than "Tooth erosion" as the translation form.	Tooth wear

Table S4. The Chinese version ACORN(CYX) scale modified after following the original author's comments on Figure S2

条目	护生口腔保健态度与信心量表
Q1	确保患者保持良好的口腔卫生是护士的职责。
Q2	患者的口腔护理与提供受压部位皮肤护理一样重要。
Q3	对于护士而言，在必要时启动牙科转诊非常重要。
Q4	护士有能力在早期发现患者的口腔健康问题。
Q5	对于吞咽困难的患者始终需要提供口腔护理。
Q6	提醒患者注意口腔保健是护士的职责。
Q7	护士应掌握进行口腔健康评估的技能。
Q8	确保所有年龄段的患者获得足够的口腔护理非常重要。
Q9	与患者讨论口腔健康问题。
Q10	与患者讨论口腔健康与自身健康状况（可能存在的问题）之间的关系。
Q11	为意识清醒但无法下床的患者提供全面的口腔护理。
Q12	在您进行口腔健康评估后，必要时将患者转诊至口腔诊疗机构。
Q13	健康的牙龈
Q14	健康的牙齿
Q15	龋齿
Q16	口腔疼痛
Q17	唇部溃疡
Q18	食物残渣
Q19	牙菌斑
Q20	牙石（牙结石）/牙垢
Q21	牙龈萎缩
Q22	牙龈发炎（如肿胀）
Q23	断牙
Q24	牙齿磨损

Table S5. Demographic characteristics of the participants in pilot testing (n=20)

Variables	Descriptor	N(n=20)	Percentage (%)
Gender	Male	8	40.00
	Female	12	60.00
Age	20	3	15.00
	21	12	60.00
	22	5	25.00
Year level (4-year study)	Year 2	2	10.00
	Year 3	18	90.00
Have taken oral care training courses or not	Yes	20	100.00
	No	0	0

Table S6. The results from Pilot Testing

Items in the ACORN(CYX) scale		Percentage of "Yes"（%）	Feedback	Revisions Made
1.	确保患者保持良好的口腔卫生是护士的职责。	100	/	/
2.	患者的口腔护理与提供受压部位皮肤护理一样重要。	100	/	/
3.	对于护士而言，在必要时启动牙科转诊非常重要。	80	What is the definition of dental referrals ?	Added definition of dental referrals.
4.	护士有能力在早期发现患者的口腔健康问题。	100	/	/
5.	对于吞咽困难的患者始终需要提供口腔护理。	100	/	/
6.	提醒患者注意口腔保健是护士的职责。	100	/	/
7.	护士应掌握进行口腔健康评估的技能。	100	/	/
8.	确保所有年龄段的患者获得足够的口腔护理非常重要。	100	/	/

9.	与患者讨论口腔健康问题。	100	/	/
10.	与患者讨论口腔健康与自身健康状况（可能存在的问题）之间的关系。	100	/	/
11.	为意识清醒但无法下床的患者提供全面的口腔护理。	100	/	/
12.	在您进行口腔健康评估后，必要时将患者转诊至口腔诊疗机构。	100	/	/
13.	健康的牙龈	100	/	/
14.	健康的牙齿	100	/	/
15.	龋齿	100	/	/
16.	口腔疼痛	100	/	/
17.	唇部溃疡	100	/	/
18.	食物残渣	100	/	/
19.	牙菌斑	90	What is the definition of plaque ?	Added definition of plaque.
20.	牙石（牙结石）/牙垢	95	What is the definition of tartar ?	Added definition of tartar.
21.	牙龈萎缩	95	What is the definition of receding gums ?	Added definition of receding gums.
22.	牙龈发炎（如肿胀）	100	/	/
23.	断牙	100	/	/
24.	牙齿磨损	95	What is the	Added definition

		definition of worn-down teeth ?	of worn-down teeth.

Table S7. Demographic characteristics of involved experts for expert consultation (n=10)

**Variables**	**Descriptor**	**N(n=10)**	**Percentage (%)**
Gender	Male	1	10.00
	Female	9	90.00
Age	30-40	6	60.00
	41-50	4	40.00
Educational background	Bachelor	4	40.00
	Master	6	60.00
	Doctor	0	0
Identity	Clinical nursing	6	60.00
	Nursing education	3	30.00
	Nursing research	1	10.00
Job title	Nurse Director	1	10.00
	Nurse Deputy Director	1	10.00
	Nurse in Charge	8	80.00
	Senior Nurse	0	0
	Others	0	0
Working experience	≤5 years	0	0
	6-10 years	1	10.00
	11-20 years	5	50.00
	>20 years	4	40.00

Table S8. The results from expert consultation

**Scale applied for expert consultation**	**Feedback**	**Revisions Made**
1. 确保患者保持良好的口腔卫生是护士的职责。	/	/
2. 患者的口腔护理与提供受压部位皮肤护理一样重要。	What is the definition of pressure area care?	Added definition of "pressure area care".
3. 对于护士而言，在必要时建议牙科转诊非常重要。（根据病情需要，建议本单位诊疗的病人转到牙科诊疗或处理）	Chinese nurses cannot directly provide dental referrals.	Added the word "recommend" before " dental referrals”.
4. 护士有能力在早期发现患者的口腔健康问题。	/	/
5. 对于吞咽困难的患者始终需要提供口腔护理。	/	/
6. 提醒患者注意口腔保健是护士的职责。	/	/
7. 护士应掌握进行口腔健康评估的技能。	/	/
8. 确保所有年龄段的患者获得足够的口腔护理非常重要。	/	/

9.	与患者讨论口腔健康问题。	/	/
10.	与患者讨论口腔健康与自身健康状况/可能存在的健康问题之间的关系。	/	/
11.	为意识清醒但无法下床的患者提供全面的口腔护理。	/	/
12.	在您进行口腔健康评估后，必要时建议患者转诊至口腔诊疗机构。	/	/
13.	健康的牙龈	/	/
14.	健康的牙齿	What is the definition of healthy teeth?	Added definition of "healthy teeth".
15.	龋齿	/	/
16.	口腔疼痛	What is the definition of oral pain?	Added definition of "oral pain".
17.	口腔溃疡	"Oral ulcer" is better expressed than "lip ulcer".	Modified "lip ulcer" to "oral ulcer" .
18.	食物残渣	/	/
19.	牙菌斑（牙菌斑指黏附在牙齿表面或口腔其他软组织上的微生物群）	/	/
20.	牙石（牙结石）/牙垢（牙石是由牙面上矿化的菌斑和其他沉积物形成的）	/	/
21.	牙龈萎缩（牙龈萎缩指牙龈边缘逐渐向根尖退缩）	/	/
22.	牙龈发炎（如牙龈肿胀）	/	/
23.	断牙	/	/
24.	牙齿磨损（牙齿磨损指主要由机械摩擦作用造成的牙体硬组织渐进性丧失的疾病）	/	/

Table S9. Demographic characteristics of participants in bilingual testing (n=10)

Variables	Descriptor	N(n=10)	Percentage (%)
Gender	Male	0	0
	Female	10	100.00
Age	21	7	70.00
	22	3	30.00
Year level(4-year study)	Year 3(4-year study)	10	100.00
Have taken oral care training courses or not	Yes	10	100.00
	No	0	0

Table S10. Scale applied for bilingual testing

Scale applied for bilingual testing

Q1	It is a nursing responsibility to ensure patients have good oral hygiene.
Q2	Providing patients' oral care is as important as pressure area care, such as preventing pressure injuries.
Q3	It is important for nurses to actively recommend dental referrals for patients when necessary. (Based on the patient's condition, you should recommend patients under your care to dental services for treatment.)
Q4	Nurses are in a good position to detect oral health problems early.
Q5	Providing oral care is always required for patients with dysphagia.
Q6	It is nurses' duty to remind patients of their oral health care.
Q7	Nurses should have the skills to conduct oral health assessments.
Q8	It is important to ensure that patients of all ages receive adequate oral care.
Q9	Discussing oral health with patients.
Q10	Discussing with patients the relationship between oral health and their overall health status or potential health issues.
Q11	Providing comprehensive oral care for patients who are conscious but unable to get out of bed.
Q12	Recommending referring patients to dental services after your oral health assessment.
Q13	Healthy gums/gingiva
Q14	Healthy teeth: teeth with intact structure and function.
Q15	Dental caries
Q16	Oral pain: pain in the mouth caused by various reasons.
Q17	Oral ulcers
Q18	Food granule
Q19	Dental plaque: a microbial community that adheres to the surface of teeth or other soft tissues in the mouth.
Q20	Dental calculus/Tartar: tartar is formed by the mineralization of plaque and other deposits on the tooth surface.
Q21	Receding gums: the gradual retreat of the gum margin towards the root apex.
Q22	Inflamed gums (e.g. swollen gums)
Q23	Broken teeth
Q24	Tooth wear: a condition characterized by the progressive loss of hard dental tissues, primarily caused by mechanical friction.
Q25	护士有能力在早期发现患者的口腔健康问题。
Q26	提醒患者注意口腔保健是护士的职责。
Q27	对于护士而言，在必要时建议牙科转诊非常重要。（根据病情需要，建议本单位诊疗的病人转到牙科诊疗或处理）
Q28	患者的口腔护理与提供受压部位皮肤护理（如预防压力性损伤）一样重要。
Q29	对于吞咽困难的患者始终需要提供口腔护理。
Q30	确保所有年龄段的患者获得足够的口腔护理非常重要。
Q31	护士应掌握进行口腔健康评估的技能。
Q32	确保患者保持良好的口腔卫生是护士的职责。
Q33	与患者讨论口腔健康与自身健康状况/可能存在的健康问题之间的关系。
Q34	在您进行口腔健康评估后，必要时建议患者转诊至口腔诊疗机构。

Q35	为意识清醒但无法下床的患者提供全面的口腔护理。
Q36	与患者讨论口腔健康问题。
Q37	牙齿磨损（牙齿磨损指主要由机械摩擦作用造成的牙体硬组织渐进性丧失的疾病。）
Q38	口腔疼痛（各种原因造成的口腔疼痛）
Q39	断牙
Q40	口腔溃疡
Q41	龋齿
Q42	食物残渣
Q43	牙菌斑（牙菌斑指黏附在牙齿表面或口腔其他软组织上的微生物群）
Q44	健康的牙齿（牙齿结构与功能完整）
Q45	健康的牙龈
Q46	牙龈发炎（如牙龈肿胀）
Q47	牙石（牙结石）/牙垢（牙石是由牙面上矿化的菌斑和其他沉积物形成的）
Q48	牙龈萎缩（牙龈萎缩指牙龈边缘逐渐向根尖退缩）

Table S11. Results from bilingual testing

**Test**	**Results**
Pearson correlation tests	Pearson correlation coefficient of 0.76 ($p < 0.01$)
paired T-tests	$p < 0.01$
ANOVA	F = 83.07 ($p < 0.01$)

Table S12. Demographic characteristics of all participants in this study (n=536)

**Variables**	**Descriptor**	**N(n=536)**	**Percentage (%)**
City	Chengdu	298	70.08
	Shenyang	238	29.92
Gender	Male	147	27.43
	Female	389	72.57
Age	19	38	7.09
	20	124	23.13
	21	161	30.04
	22	146	27.24
	23	58	10.82
	24	9	1.68
Year level（4-year study）	Year 2	134	25.00
	Year 3	185	34.51
	Year 4	217	40.49
Have taken oral care training courses or not	Yes	536	100.00
	No	0	0

Table S13. Item analysis of the Chinese version of ACORN(CYX)

Item	Item content	low-score group （M±SD）	high-score group （M±SD）	CR(t value)/p value	r value/p value
Q1	It is a nursing responsibility to ensure patients have good oral hygiene.	4.86±1.43	6.59±0.59	13.34/p<0.01	0.60/p<0.01
Q2	Providing patients' oral care is as important as pressure area care, such as preventing pressure injuries.	4.79±1.41	6.66±0.50	14.94/p<0.01	0.61/p<0.01
Q3	It is important for nurses to actively recommend dental referrals for patients when necessary. (Based on the patient's condition, you should recommend patients under your care to dental services for treatment.)	4.91±1.29	6.62±0.53	14.71/p<0.01	0.61/p<0.01
Q4	Nurses are in a good position to detect oral health problems early.	4.72±1.20	6.51±0.58	15.98/p<0.01	0.62/p<0.01
Q5	Providing oral care is always required for patients with dysphagia.	4.94±1.30	6.56±0.71	13.12/p<0.01	0.56/p<0.01
Q6	It is nurses' duty to remind patients of their oral health care.	4.85±1.40	6.65±0.53	14.37/p<0.01	0.61/p<0.01
Q7	Nurses should have the skills to conduct oral health assessments.	5.08±1.23	6.69±0.49	14.66/p<0.01	0.60/p<0.01
Q8	It is important to ensure that patients of all ages receive adequate oral care.	4.85±1.22	6.62±0.50	15.95/p<0.01	0.65/p<0.01
Q9	Discussing oral health with patients.	4.19±1.17	6.49±0.62	20.84/p<0.01	0.75/p<0.01
Q10	Discussing with patients the relationship between oral health and their overall health status or potential health issues.	4.50±1.17	6.48±0.60	18.04/p<0.01	0.69/p<0.01
Q11	Providing comprehensive	4.70±1.18	6.41±0.68	14.95/p<0.01	0.62/p<0.01

	oral care for patients who are conscious but unable to get out of bed.				
Q12	Recommending referring patients to dental services after your oral health assessment.	4.34±1.23	6.44±0.63	18.19/p<0.01	0.69/p<0.01
Q13	Healthy gums/gingiva	4.47±1.25	6.45±0.68	16.69/p<0.01	0.71/p<0.01
Q14	Healthy teeth: teeth with intact structure and function.	4.50±1.30	6.63±0.54	18.04/p<0.01	0.71/p<0.01
Q15	Dental caries	4.11±1.38	6.53±0.67	18.86/p<0.01	0.70/p<0.01
Q16	Oral pain: pain in the mouth caused by various reasons.	3.81±1.25	6.34±0.81	20.31/p<0.01	0.73/p<0.01
Q17	Oral ulcers	4.18±1.32	6.64±0.52	20.77/p<0.01	0.76/p<0.01
Q18	Food granule	4.37±1.35	6.53±0.64	17.29/p<0.01	0.68/p<0.01
Q19	Dental plaque: a microbial community that adheres to the surface of teeth or other soft tissues in the mouth.	3.82±1.24	6.51±0.63	23.14/p<0.01	0.78/p<0.01
Q20	Dental calculus/Tartar: tartar is formed by the mineralization of plaque and other deposits on the tooth surface.	3.97±1.32	6.50±0.60	20.90/p<0.01	0.77/p<0.01
Q21	Receding gums: the gradual retreat of the gum margin towards the root apex.	3.82±1.30	6.44±0.70	21.30/p<0.01	0.74/p<0.01
Q22	Inflamed gums (e.g. swollen gums)	4.01±1.29	6.45±0.62	20.34/p<0.01	0.73/p<0.01
Q23	Broken teeth	4.22±1.41	6.58±0.53	18.78/p<0.01	0.72/p<0.01
Q24	Tooth wear: a condition characterized by the progressive loss of hard dental tissues, primarily caused by mechanical friction.	3.88±1.24	6.47±0.69	21.90/p<0.01	0.75/p<0.01
Total	/	105.88±15.37	156.80±5.81	/	/
